# Impact of geometric uncertainties on dose distribution during intensity modulated radiotherapy of head-and-neck cancer: the need for a planning target volume and a planning organ-at-risk volume

**DOI:** 10.3390/curroncol13030010

**Published:** 2006-06

**Authors:** O. Ballivy, W. Parker, T. Vuong, G. Shenouda, H. Patrocinio

**Affiliations:** * Division of Radiation Oncology, Department of Oncology, McGill University, Montreal, Quebec; † Medical Physics Unit, Department of Oncology, McGill University, Montreal, Quebec

**Keywords:** imrt, ptv margins, head and neck, geometric uncertainties, 3d crt

## Abstract

We assessed the effect of geometric uncertainties on target coverage and on dose to the organs at risk (oars) during intensity-modulated radiotherapy (imrt) for head-and-neck cancer, and we estimated the required margins for the planning target volume (ptv) and the planning organ-at-risk volume (prv). For eight head-and-neck cancer patients, we generated imrt plans with localization uncertainty margins of 0 mm, 2.5 mm, and 5.0 mm. The beam intensities were then applied on repeat computed tomography (ct) scans obtained weekly during treatment, and dose distributions were recalculated.

The dose–volume histogram analysis for the repeat ct scans showed that target coverage was adequate (V_100_ ≥ 95%) for only 12.5% of the gross tumour volumes, 54.3% of the upper-neck clinical target volumes (ctvs), and 27.4% of the lower-neck ctvs when no margins were added for ptv. The use of 2.5-mm and 5.0-mm margins significantly improved target coverage, but the mean dose to the contralateral parotid increased from 25.9 Gy to 29.2 Gy. Maximum dose to the spinal cord was above limit in 57.7%, 34.6%, and 15.4% of cases when 0-mm, 2.5-mm, and 5.0-mm margins (respectively) were used for prv.

Significant deviations from the prescribed dose can occur during imrt treatment delivery for head-and-neck cancer. The use of 2.5-mm to 5.0-mm margins for ptv and prv greatly reduces the risk of underdosing targets and of overdosing the spinal cord.

## 1. INTRODUCTION

Intensity modulated radiotherapy (imrt) uses an optimized modulation of the beam fluences to generate complex dose distributions that are characterized by a high degree of conformity to the target volume and greater sparing of normal tissue. In the treatment of head-and-neck cancer, imrt is used mainly to preserve salivary function after radiotherapy 1–4 and to improve dose coverage for complexly shaped tumours located near critical structures 5–7. In addition, because imrt allows a higher dose to be delivered to the macroscopic tumour than to the area at risk for microscopic disease within the same fraction, the imrt technique is ideally suited to the simultaneous delivery of integrated-boost accelerated fractionation schemes 8,9.

Accounting for geometric uncertainties is an important issue with imrt, because the isodose lines conform tightly to the target volume. One approach to compensate for set-up errors, organ motion, and changes in target geometry consists of defining planning target volumes (ptv) for targets and planning organ-at-risk volumes (prv) for critical structures, as recommended by the International Commission on Radiation Units and Measurements (icru) 10,11. For the head-and-neck region, the safety margins included in the ptv are usually chosen to reflect expected setup uncertainties.

During treatment, portal imaging can be used to assess the accuracy of field alignment. However, acquiring port films for imrt is often impractical because of the complex modulation of the beam intensity. Thus, set-up verification for imrt usually consists of acquiring orthogonal images to check the isocentre localization. Verification methods that rely on this kind of two-dimensional imaging may not provide a comprehensive overview of all the geometric variations that can influence dose distribution in an imrt treatment. Slight variations in the patient’s neck and shoulder positioning may not be properly assessed, and some significant changes in shape because of a patient’s weight loss or tumour shrinkage may not be detected. Furthermore, the effects of these changes on the dose distribution are not as intuitive during treatment with imrt techniques. A proper ptv and prv definition for imrt should not only focus on the potential location of the target and the organs at risk during treatment, but should also integrate any geometric variation in the surrounding tissue that may result in significant deviation from the prescribed dose.

In the present study, we assessed the effect of geometric variations—including changes in patient anatomy and set-up uncertainties—on the target coverage and the dose to organs at risk by recalculating imrt dose distributions on repeat computed tomography (ct) scans taken during treatment. In addition, we generated imrt plans using 2.5-mm and 5.0-mm localization uncertainty margins to estimate the ptv and prv that would be effective in preventing significant underdosing of the target volume and overdosing of the critical structures.

## 2. PATIENTS AND METHODS

### 2.1 Patient Characteristics

We included 8 head-and-neck cancer patients consecutively treated with imrt at our institution in this dosimetric study. Primary tumour sites included oropharynx (4 patients), nasopharynx (2 patients), maxillary sinus (1 patient), and nodal metastasis from an unknown primary (1 patient). All patients had locally advanced stage iii–iv(a) disease according to the 2002 American Joint Committee on Cancer classification. In 7 patients, the treatment volume consisted of comprehensive nodal irradiation (upper and lower neck nodes), and 1 patient received treatment to the primary tumour site and upper neck region only. Two patients also received concurrent chemotherapy with cisplatin.

### 2.2 CT Scans

Two weeks before the start of treatment, all patients first underwent ct simulation in the treatment position. To ensure proper immobilization and treatment reproducibility, custom-made thermoplastic face masks and shoulders pulls were used. The slice thickness of the ct scan was 5 mm through the entire head and neck region. Each patient then underwent repeat ct scans at weekly intervals during treatment, with the goal of obtaining at least four ct scans per patient. However, because of severe weight loss (3 patients) or significant shrinkage of a large neck mass (1 patient), some patients required a new mask during the course of treatment. In each of these cases, a new imrt plan was generated for the remainder of the treatment. As a result, only the repeat ct scans that were made with the initial mask were used for the study. Thus, 4 patients had four repeat ct scans as planned, 2 patients had three ct scans, and 2 patients had two ct scans, for a total of 26 repeat ct scans. All the repeat ct scans were acquired using the same ct protocol that was used in the planning ct scans and radio-opaque markers were placed on the thermoplastic masks to indicate the treatment isocentre.

### 2.3 Contouring Target Volumes and OAR

The gross tumour volume (gtv), clinical target volume (ctv), and organs at risk (oars) were contoured on the initial planning ct scan according to icru definitions. The primary tumour and each positive node were delineated as separate volumes, except when they were immediately adjacent. The ctv was defined as any potential area of microscopic disease and usually included a margin of at least 1 cm around the gtv to account for direct tumour invasion (except when it was adjacent to structures known not to be involved), plus the lymph-node regions at risk. For dose prescription and dvh analysis, the upper and lower nodal neck regions were contoured separately. The normal structures that were defined were the spinal cord and brain stem, the ipsilateral and contralateral parotids, the mandible, and the larynx. The target volumes (gtv and ctv) and oars were then redrawn on all the repeat ct scans by a single radiation oncologist. An anatomy-matching fusion algorithm semiautomatically matched the original with the repeat CT scans so that the original targets were superimposed on the repeat CT scans. The target contours were then modified with respect to changes in patient anatomy.

### 2.4 IMRT Treatment Planning

The ct images and radiotherapy structures were transferred from the initial planning ct scans to an inverse planning system (Corvus 5.0: Nomos Corporation, Sewickley, PA, U.S.A.), and imrt plans were generated using 7–9 equally spaced 6-MV photon beams. The prescribed dose was 66–70 Gy to the gtvs, 54–60 Gy to the upper neck ctv, and 54 Gy to the lower neck ctv, planned for delivery in a single phase using fractions of 1.6–2 Gy for the ctvs and 2.12–2.4 Gy for the gtvs. The planning goal was to provide adequate target coverage (prescription isodose encompassing at least 95% of each target) while limiting the dose to the oars (spinal cord: <45 Gy; brain stem: <54 Gy; parotid mean dose: <26 Gy).

For each patient, three imrt plans were generated: one with no margins for localization uncertainty, and two with margins (2.5-mm, 5.0-mm) around the gtv and ctv (resulting in ptvs) and around the spinal cord and brain stem (prvs). No margins were added around the parotids and the other oars.

### 2.5 Recalculation of Dose Distributions and Analysis

The beam configurations and intensity profiles of the initial imrt plans generated from the planning ct scans were then applied on each repeat ct scan. The radio-opaque fiducials placed on the thermoplastic mask were used for isocentre alignment. Dose distributions were recalculated, and dose–volume histograms (dvhs) were generated for the targets and oars. To assess dose coverage, the dosimetric parameters that were analyzed included the percentage of the ptv that received at least 100% and 95% of the prescribed dose (V_100_, V_95_). In addition, the lowest dose to 1% of the target volume, which can also be defined as the dose delivered to at least 99% of the volume (D_99_), was used to monitor for cold spots inside the target volume.

For the spinal cord and brain stem, the maximum dose (D_max_) and the highest dose to a volume of 1 cm^3^ (D_1cm3_) were analyzed. The mean doses (D_mean_) to the ipsilateral and contralateral parotids were used to assess parotid sparing, and the D_mean_ to the larynx, plus the D_1cm3_ to the mandible were also recorded.

## 3. RESULTS

### 3.1 Analysis of the Target Coverage

Table I summarizes the analysis of the target coverage for the initial imrt plans and for the recalculated dose distributions from the repeat ct scans. Note that, for the initial imrt plans, the values reported are for the ptvs generated with 0-mm, 2.5-mm, and 5.0-mm uncertainty margins, and for the repeat ct scans, values are for the gtvs and ctvs.

#### 3.1.1 Initial IMRT Plans

The coverage of the ptvs as assessed by the mean values of V_100_ and V_95_ was similar for the initial imrt plans generated without any margins and for plans with 2.5-mm and 5.0-mm margins. However, the mean value of D_99_ was lower for the upper- and lower- neck ptvs when 5-mm margins were used. This difference probably arose because portions of these larger ptvs overlapped with the surrounding normal structures (such as the parotid glands), resulting in a compromise in target coverage to achieve adequate organ sparing. In addition, the ptvs generated by adding 5.0-mm margins around the nodal-region ctvs frequently extended into the build-up region close to the skin surface, where the calculated dose may be lower.

#### 3.1.2 Repeat CT Scans

**Figure 1:**
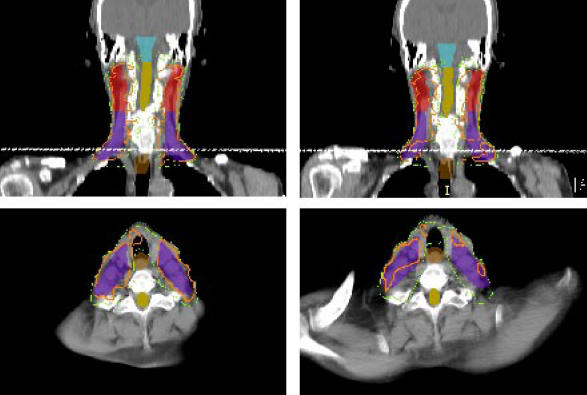
For this patient, elevation of the shoulder during the repeat computed tomography (ct) scan (right) as compared with the planning ct scan (left) resulted in reduced coverage of the lower neck region because of greater attenuation of the oblique beams. The effect on the prescription isodose (orange) is significant, but influence on the coverage by the 90% isodose (green) is minimal.

Overall, when no localization uncertainty margins were added at the time of planning, the mean values of V_100_, V_95_, and D_99_ were significantly lower for the repeat ct scans than for the initial imrt plans. The dose coverage improved for all target volumes when ptvs were generated. Most of the benefit was seen with the use of the 2.5-mm margins; the 5.0-mm margins resulted in modest further improvement. The rate of adequate target coverage, as defined by the proportion of targets with a V_100_ greater than or equal to 95% also improved with the use of ptvs. When no margins were added for uncertainty, only 12% of the gtvs, 54% of the upper-neck ctvs, and 25% of the lower-neck ctvs had a V_100_ ≥ 95%. With the use of 2.5-mm and 5.0-mm margins, this proportion increased to 87.5% and 97.5% for the gtvs, and to 97.1% and 100% for the upper-neck ctvs respectively. However, with the 2.5-mm and 5.0-mm margins alike, only 72.7% of the lower-neck ctvs were adequately covered by the prescription isodose. This result reflects significant variation in shoulder position at the time of the repeat ct scan from the position at the initial planning ct scan. As shown in Figure 1, the elevation of the shoulder on the ct scan acquired during week 2 caused a major change in the shape of the external contour at that level, resulting in increased attenuation of the oblique beams. However, the impact of positioning errors on coverage by the 95% isodose was not as pronounced, and when 5.0 mm margins where used, the V_95_ for the lower-neck ctv was greater than or equal to 95% in all cases.

### 3.2 Analysis of the Dose to the OARs

Table II summarizes the analysis of the dose to the oars for the initial imrt plans and of the recalculated dose distributions from the repeat ct scans. Note that, for the initial imrt plans, the values of D_max_ and D_1cm3_ for the spinal cord and brain stem are for the prvs generated with 0-mm, 2.5-mm, and 5.0-mm localization uncertainty margins; for the repeat ct scans, the values are for the anatomic structures only.

#### 3.2.1 Initial IMRT Plans

The average D_max_ to the spinal cord and brain stem prvs was slightly above the specified limit for the initial imrt plans generated with 2.5-mm and 5.0-mm localization uncertainty margins. However, the dose to the anatomic structures (without margins) was below 45 Gy for the spinal cord and 54 Gy for the brain stem in all plans.

Unsurprisingly, the D_mean_ to the parotids and larynx, and the D_1cm3_ to the mandible increased when 2.5-mm and 5.0-mm margins were added around the target volumes to generate ptvs.

#### 3.2.2 Repeat CT Scans

The D_max_ to the spinal cord was greater than 45 Gy in 57% of repeat ct scans when no prv was used, as compared with 35% with 2.5-mm margins and 15.4% with 5.0-mm margins. However, the D_max_ to the brain stem was above 54 Gy in only 3.8% of the cases when no prv was generated and in 7.8% of the cases when 2.5-mm and 5.0-mm margins were added. A possible explanation is that the prvs were generated only when similar margins were also added for the ptv. Because of the narrower separation between the target volume and the brain stem when 2.5-mm and 5.0-mm margins were added, the 54 Gy isodose was closer to the brain stem for the plans with ptv and prv than for the plans with no margins. The highest D_1cm3_ to both the spinal cord and the brain stem were below 45 Gy and 54 Gy, respectively, for all repeat ct scans.

The D_mean_ to the parotids and larynx and the D_1cm3_ to the mandible were also higher for the recalculated dose distributions than for the imrt plans. The average increase in the D_mean_ was 1–1.5 Gy for the contralateral parotid, but 2–3 Gy for the ipsilateral parotid. The magnitude of the increase was similar for all plans whether generated with 0-mm, 2.5-mm, or 5.0-mm localization uncertainty margins.

## 4. DISCUSSION

As compared with conventional portal images, ct scan imaging for treatment verification provides a more accurate representation of the geometric variations that occur during a course of head-and-neck imrt, including uncertainties in patient position, isocentre localization, organ motion, and changes in external contour because of weight loss or tumour shrinkage. The anatomic information from the ct scan can also be used to generate three-dimensional (3-D) dose distributions to estimate with greater accuracy the dose delivered to the targets and the critical structures during a fractionated course of imrt. However, such evaluation is labour-intensive and not without inconveniences.

First, delineation of target volumes and oars may not be identical for the treatment planning ct scan and each of the repeat ct scans acquired during treatment. A recent study by Barker *et al.* 12 showed that the gtvs decreased in size throughout the course of treatment at a median rate of nearly 2% per treatment day. Contouring variations or changes in tumour shape or size may thus influence the dvhs for these structures.

In our study, image-fusion techniques and standardized target volume definitions based on well-defined anatomic landmarks 13,14 were used to minimize contouring discrepancies between the initial planning ct scan and the repeat ct scans. As shown in Table III, only small variations in the size of the gtvs and ctvs occurred, except in the case of a few positive nodes that shrank during treatment. Also, the use of dosimetric parameters alone to assess target coverage and organ sparing may not be sufficient for imrt dose distributions. Analyzing dvhs does not give information about whether an area of lower dose is localized in the periphery or in the central region of the target volume, or if an area of higher dose represents a clinically significant hot spot within a critical structure or instead consists of multiple hot foci of negligible size. In addition, it is impossible to tell if areas of dose perturbation are always located at the same place on each of the ct scans.

However, despite these limitations, the use of ct imaging together with 3-D dosimetric analysis constitutes an effective method to study the effects of geometric uncertainties on the dose.

Adding margins for localization uncertainty at the time of treatment planning can effectively reduce the hazardous effect of these variations on target coverage and dose to critical structures. However, the addition of margins may not compensate completely for dose perturbation caused by a significant change in external contour.

In fact, redesign or modification of the ptv cannot compensate for the underdosing in the lower neck region observed when the shoulders were not properly repositioned at the time of the repeat ct scan. The effect of less radical changes in the shape of the external contour, such as those related to weight loss or tumour shrinkage, are probably not as important. A dosimetric study by Kim *et al.* 15 showed that expansion and contraction of the external contour by 5 mm in all directions for a head-and-neck imrt plan does not result in significant dose perturbation for the tumour and critical structures.

Our results also suggest that the amplitude of the ptv required to assure ideal target coverage during treatment may vary, depending on target volume location, size, and shape. In our study, adding 2.5-mm localization uncertainty margins appeared sufficient to provide good target coverage more than 95% of the time (V_100_ ≥ 95%) for the upper neck ctv; 5.0-mm margins were required to achieve the same level of coverage for gtvs. This finding may be explained by the fact that a given set-up error—a 3-mm misalignment of the isocentre, for example—will have a greater influence on the target coverage (as assessed by the V_100_) for a positive lymph node measuring 1 cm than for the entire upper-neck region. In addition, set-up accuracy is rarely uniform through the whole treatment volume; the importance of the geometric uncertainty may vary depending on the location of the target.

In a study by Gilbeau *et al.* 16, reproducibility of the set-up was found to be significantly worse at the level of the shoulder with the use of a thermoplastic mask that covers the head only. A similar observation was made by Garg *et al.* 17, who reported that, despite custom immobilization, the curvature of the cervical spinal changes during the course of treatment, and set-up reproducibility was inferior for isocentres located in the low-neck region. Additional margins for organ motion may also be required for tumours involving the base of the tongue or the larynx 18, but such margins are probably unnecessary for a nasopharyngeal tumour.

Proper ptv margins should not only focus on target coverage, but also consider the effect on normal-tissue sparing. Our results showed that the D_mean_ to the parotids and larynx increases when larger margins are added for ptv at the time of planning. Similar observations have also been made by van Asselen *et al.* 19, who reported that the use of large ptv margins reduces parotid sparing and increases the normal-tissue complication probability for xerostomia. Geometric variations between the planning ct scans and the repeat ct scans also seem to influence the mean dose to the parotids in a similar manner, with the greater increase being observed when 5.0-mm localization uncertainty margins are added around the upper-neck ctv. Manning *et al.* 20 proposed the use of a prv for the contralateral parotid to compensate for the detrimental effect of the geometric variation on parotid sparing. Their work suggests that 5.0-mm margins were effective in preventing the increase in the contralateral parotid volume irradiated to 30 Gy when the isocentre was shifted toward that side.

In addition to physical position uncertainties, other geometric variations, such as changes in the volume of the glands during treatment, may also influence the dose delivered to the parotids. In the study by Barker *et al.* 12, the parotid glands decreased in volume with time, at a rate of 0.6% per day, and the centre of the glands shifted medially because of the weight loss that occurred during treatment. Thus, simply adding prv margins around the parotids may not compensate properly for the overall effect of geometric uncertainties on the D_mean_ to the parotids. Moreover, in the study by Manning *et al.* 20, the use of 5-mm prv margins around the contralateral parotids resulted in decreased coverage of the nodal region adjacent to the parotids. To avoid this problem and to limit planning complexity, we consider that prv should be used only for structures such as the brain stem and spinal cord, for which the consequence of overdosing may be serious.

In the present study, the selection of 2.5-mm and 5.0-mm margins was based mainly on the range of the set-up errors reported for head-and-neck cancer patients treated with custom immobilization devices 15,16,21–26. Our results suggest that these margins appear to give satisfactory target coverage, but care must be taken when generalizing about required margins for ptv and prv. Because the repeat ct scans were not acquired with the patient lying on the treatment couch, this study did not assess some of the mechanical uncertainties inherent to imrt treatment delivery. Thus, we cannot assume that the variations in set-up between the planning ct and each repeat ct scan are truly representative of those encountered at the treatment machine. Uncertainties in couch and gantry geometry or in alignment of the lasers relative to the treatment machine isocentre may effectively introduce systematic errors between the ct planning and treatment machine set-up 27. The integrated ct/linac unit 28,29 now allows for 3-D imaging verification at the treatment machine, and such systems may be used for future investigations.

Our study did not address the issue of the intrafractional variations attributable to patient and internal-organ motion. However, in a study by Lee *et al.* 30, 20 patients undergoing imrt for head-and-neck cancer had a set of orthogonal portal images taken before and after each fraction of radiotherapy for 3 consecutive days. Overall, the magnitude of intrafraction patient motion was minimal (x axis: 0.4 mm + 1.2 mm; y axis: 1.2 mm + 1.6 mm; z axis: –0.6 mm + 1.2 mm), and no persistent intrafraction motion was observed. In another study using an infrared camera monitoring system, Kim *et al.* 31 also found that intrafraction positioning uncertainty was less than 2 mm for head-and-neck cancer patients treated with imrt. Based on these results, intrafraction patient motion does not seem to differ significantly from interfraction set-up uncertainty.

Finally, our results are based on a relatively low number of repeat ct scans per patient (median: 3). For that reason, dvhs from the 26 ct scans obtained for the 8 patients were pooled to assess target coverage and dose to oars. This population-based approach may not reflect recurrent systematic deviations in some patients 32. In our study, 2 patients had repeated errors in the positioning of the shoulder, which caused underdosing of the lower neck region; coverage for the other patients was adequate when 2.5-mm and 5.0-mm margins were used. Additionally, because only the repeat ct scan acquired with the same mask and immobilization system as that used for the initial planning ct scan were included in our analysis, we cannot presume that our findings apply to patients with severe weight loss or tumour shrinkage that produces changes in the surrounding tissue important enough to affect the quality of the immobilization. At our institution, a new mask is made whenever the mask does not fit properly or the isocentre does not align correctly on repeated portal verifications. One of the problems with trying to adjust patient position to fit more tightly within the mask is that these attempts may modify the geometry of the entire neck region relative to the isocentre, thus resulting in misalignment of the beams in the some part of the treated volume despite a somewhat adequate isocentre alignment.

## 5. CONCLUSIONS

In the present investigation, the beam intensities of initial imrt plans were applied on repeat ct scans obtained weekly during treatment to assess the impact of geometric uncertainties on the dose distributions for head-and-neck cancer patients treated with imrt. Significant deviation from the prescribed dose occurred during fractionated imrt treatment because of variations in patient positioning and changes in the surrounding tissue. Adding localization uncertainty margins at the time of planning to generate ptvs and prvs reduces the risk of underdosing targets and overdosing the spinal cord. However, the D_mean_ to both parotids and to the larynx tends to increase with the size of the margins added for ptv. Based on our findings, use of 5.0-mm ptv margins for the gtv and 2.5-mm ptv margins for the upper neck ctv achieves a satisfactory compromise between target coverage and parotid sparing.

Our results also show that, for patients treated with comprehensive nodal imrt, errors in shoulder repositioning can cause significant dose perturbations in the lower neck region despite the use of 5.0-mm ptv margins. The use of a thermoplastic mask extending down to the level of the shoulder should be considered in these patients to improve set-up reproducibility.

Finally, the concept of ptv and prv will certainly evolve with the use of inverse planning methods for imrt. Integration of the geometric uncertainty in the form of probabilistic algorithms in the optimization process has been proposed to maximize the probability of target coverage and oar sparing 33. Further quantification of the geometric variations and their effect on the dose distribution is essential to the development of integrated planning systems of this kind.

## Figures and Tables

**Table I:** Target coverage for the initial intensity modulated radiotherapy (imrt) plans and repeat computed tomography (ct) scans when 0-mm, 2.5-mm, and 5.0-mm localization uncertainty margins were used a

	*Margins (mean % ± standard deviation)*					
	*0 mm*		*2.5 mm*		*5.0 mm*	
*Targets*	*Initial plan*	*Repeatct*	*Initial plan*	*Repeatct*	*Initial plan*	*Repeatct*
gtv						
V_100_^b^	95.7±0.7	85.9±9.8	95.6±0.7	96.9±4.1	95.8±1.4	99.1±1.1
V_95_^b^	99.9±0.2	97.4±3.9	99.8±0.2	99.6±1.0	99.7±0.4	99.9±0.1
D_99_^c^	98.4±0.4	94.0±3.2	97.9±1.1	98.5±2.3	97.8±2.0	99.9±1.3
ctv upper neck						
V_100_^b^	97.6±0.4	94.7±2.9	97.5±1.0	98.4±1.3	95.4±1.4	98.7±1.2
V_95_^b^	99.5±0.3	97.9±1.6	99.3±0.5	99.5±0.5	98.1±0.5	99.7±0.3
D_99_^c^	97.3±1.2	91.0±5.6	96.0±3.1	98.9±2.8	91.4±3.2	99.7±2.2
ctv lower neck						
V_100_^b^	96.9±1.2	88.3±8.3	97.9±1.0	94.8±5.6	96.1±0.4	96.8±4.4
V_95_^b^	99.4±0.3	95.1±4.5	99.3±0.3	98.5±2.2	98.5±0.4	99.4±0.9
D_99_^c^	97.0±1.6	85.1±10.5	98.0±1.6	95.3±5.4	90.0±7.1	98.9±3.0

a For the initial imrt plans, values are for the planning target volume obtained by expanding the gross tumour volume (gtv) and clinical target volume (ctv) with 0 mm, 2.5 mm, and 5.0 mm localization uncertainty margins. For the repeat ct scans (recalculated dose distributions), the values are for the gtvs and ctv, given that the localization uncertainty margins were added for planning only.

b Reported as a percentage (%) of the absolute volume.

c Reported as a percentage (%) of the prescribed dose to the gtvs and ctvs.

V_100_ = 100% of the prescribed dose; V_95_ = 95% of the prescribed dose; D_99_ = the dose delivered to at least 99% of the volume.

**Table II:** Dose to organs at risk (oars) for the initial intensity modulated radiotherapy (imrt) plans and repeat computed tomography (ct) scans when 0-mm, 2.5-mm, and 5.0-mm localization uncertainty margins were used

	*Margins (mean Gy ± standard deviation)*					
	*0 mm*		*2.5 mm*		*5.0 mm*	
*oar*	*Initial plana*	*Repeatctb*	*Initial plana*	*Repeatctb*	*Initial plana*	*Repeatctb*
Spinal cord						
D_max_	43.1±1.4	45.9±3.1	45.8±0.8	44.9±2.6	46.9±0.9	43.0±3.3
D_1cm3_	37.9±1.0	38.5±2.1	41.1±0.6	38.8±1.7	42.6±1.0	38.1±2.1
Brain stem						
D_max_	40.8±8.1	42.1±7.4	49.5±3.9	45.6±5.9	55.6±2.6	48.4±4.0
D_1cm3_	33.9±8.4	34.5±8.2	41.8±5.4	35.9±6.8	49.4±3.3	41.2±4.8
Parotid contralateral						
D_mean_	24.4±0.6	25.9±4.0	25.6±0.5	26.7±4.4	27.7±1.5	29.2±5.4
Parotid ipsilateral						
D_mean_	25.8±1.6	28.1±3.3	28.6±3.8	31.4±4.3	31.2±4.5	34.0±5.4
Larynx						
D_mean_	30.6±1.5	33.4±2.7	32.4±32.4	35.3±3.8	36.9±2.5	39.3±3.6
Mandible						
D_1cm3_	60.5±3.4	61.5±3.9	63.3±3.6	64.1±4.0	66.0±2.8	66.9±3.6

a Values are for the planning organ-at-risk volume (prv) obtained by expanding the spinal cord and brain stem volume with 0 mm, 2.5 mm, and 5.0 mm localization uncertainty margins. No prvs were generated for the parotids, larynx, and mandible.

b Values are for the spinal cord and brain-stem volume; the localization uncertainty margins were added for planning only.

D_max_ = maximum dose; D_1cm3_ = the highest dose to a volume of 1 cm^3^; D_mean_ = mean dose.

**Table III:** Difference a in the gross tumour volume (gtv) and clinical target volume (ctv) between the repeat computed tomography (ct) scans and the planning ct scans

	*gtv*			
	*Primary*	*Nodal*	*Upper neckctv*	*Lower neckctv*
Mean difference (%)	+1.6±3.4	−6.0±10.0	−1.3±2.6	+1.7±4.0

a Percentage of the absolute volume, obtained by comparing the gtv and ctv volumes from the repeat ct scans with the corresponding planning ct scan.
